# Recent Aspects of Periodontitis and Alzheimer’s Disease—A Narrative Review

**DOI:** 10.3390/ijms25052612

**Published:** 2024-02-23

**Authors:** Dominika Cichońska, Magda Mazuś, Aida Kusiak

**Affiliations:** 1 Department of Periodontology and Oral Mucosa Diseases, Medical University of Gdańsk, Orzeszkowej 18 St. 18, 80-208 Gdańsk, Poland; akusiak@gumed.edu.pl; 2 Student Research Group of the Department of Periodontology and Oral Mucosa Diseases, Medical University of Gdańsk, Orzeszkowej 18 St. 18, 80-208 Gdańsk, Poland; magdamazus@gmail.com

**Keywords:** periodontitis, Alzheimer’s disease, oral microbiome, *Porphyromonas gingivalis*, lipopolysaccharides

## Abstract

Periodontitis is an inflammatory condition affecting the supporting structures of the teeth. Periodontal conditions may increase the susceptibility of individuals to various systemic illnesses, including Alzheimer’s disease. Alzheimer’s disease is a neurodegenerative condition characterized by a gradual onset and progressive deterioration, making it the primary cause of dementia, although the exact cause of the disease remains elusive. Both Alzheimer’s disease and periodontitis share risk factors and clinical studies comparing the associations and occurrence of periodontitis among individuals with Alzheimer’s disease have suggested a potential correlation between these conditions. Brains of individuals with Alzheimer’s disease have substantiated the existence of microorganisms related to periodontitis, especially *Porphyromonas gingivalis*, which produces neurotoxic gingipains and may present the capability to breach the blood–brain barrier. *Treponema denticola* may induce tau hyperphosphorylation and lead to neuronal apoptosis. Lipopolysaccharides—components of bacterial cell membranes and mediators of inflammation—also have an impact on brain function. Further research could unveil therapeutic approaches targeting periodontal pathogens to potentially alleviate AD progression.

## 1. Introduction

Periodontitis is an inflammatory condition affecting the supporting structures of the teeth, including the gums, periodontal ligament, and alveolar bone. It is characterized by the presence of gum inflammation, loss of periodontal attachment, and destruction of surrounding tissues. Untreated periodontitis leads to tooth loss, causing a serious disruption in quality of life [1]. The development of periodontitis involves a progression from an initial disturbance in the microbial balance driven by the host’s response. If the biofilm is not disrupted or removed, this initial imbalance evolves into a more severe dysbiosis, increasing an ongoing and destructive inflammatory state that does not easily resolve [2]. 

This progression is facilitated by several factors. Firstly, DAMPs (damage-associated molecular patterns) released by damaged cells activate the immune system, contributing to the development of inflammation [3]. Then, bacterial products attract immune cells, particularly neutrophils, to the site of infection or inflammation [4] and gingival crevicular fluid (GCF) containing inflammatory components flows into the periodontal pockets [5]. Lipopolysaccharide (LPS), a component of certain bacteria, triggers immune responses and inflammation [6]. Matrix metalloproteinases (MMPs) also play a significant role in tissue degradation and breakdown of the extracellular matrix, contributing to tissue damage during periodontitis development [7]. Polymorphonuclear neutrophils (PMNs), a type of white blood cell, are also involved in the inflammatory response against infection [8]. The persistent presence of microbial plaque biofilm leads to an ongoing host immune response, altering the microbial community and causing an imbalance or dysbiosis. This dysbiosis, correlated with the sustained inflammatory reaction, results in tissue damage and the loss of alveolar bone, leading to the advancement of periodontitis. The disruption of the biofilm through proper oral hygiene, including a professional dental intervention, is essential to prevent the progression of gingivitis into periodontitis [9].

Periodontal conditions may increase the susceptibility of individuals to various systemic illnesses such as cardiovascular disease [10], oral cancer and colorectal neoplasms [11,12,13], gastrointestinal issues [14], respiratory tract infections and pneumonia [15,16], adverse outcomes during pregnancy [17,18,19], diabetes [20,21,22], insulin resistance [23], and neurodegenerative disorders, including Alzheimer’s disease [24,25,26]. 

Various medications might have an impact on the evolution and treatment of periodontal disease, including anti-rheumatic therapies. Several classes of anti-inflammatory drugs recommended in rheumatoid arthritis that modulate the host response might find an application in periodontal treatment, such as corticosteroids, disease-modifying antirheumatic drugs, non-steroidal anti-inflammatory drugs, matrix metalloproteinase inhibitors, bisphosphonates, Toll-like receptor inhibitors, and others. However, a combined antibacterial and anti-inflammatory approach should be considered [27].

Alzheimer’s disease (AD) is a neurodegenerative condition characterized by a gradual onset and progressive deterioration, making it the primary cause of dementia. The most prominent symptom is a decline in short-term memory and a gradual loss of cognitive function [28].

The exact cause of AD remains elusive. Only a small fraction, around 1–2%, of AD cases are directly inherited and follow an autosomal dominant pattern. This form, known as familial AD, presents with early-onset symptoms and a swift progression of symptoms [29]. Early onset AD is commonly attributed to genetic factors, suggesting a strong hereditary component. On the contrary, late-onset or sporadic AD, which accounts for the majority of cases, is thought to arise from a complex interplay between genetics and environmental factors. Several factors have been identified as potential contributors to late-onset AD. These include a family history of the disease, education level, a diet high in fat content, high blood pressure, diabetes, a history of head injuries, and specific genetic predispositions like the presence of the APOE ε4 (Apolipoprotein E ε4) gene variant [30]. It is important to note that while early-onset AD tends to have a stronger genetic basis, late-onset AD is a multifactorial condition influenced by a combination of genetic susceptibility and environmental factors. This intricate interplay underscores the complexity of understanding and addressing the various risk factors associated with Alzheimer’s disease [31].

Both Alzheimer’s disease and periodontitis share risk factors stemming from various aspects such as oral hygiene practices, tobacco use, dietary habits, systemic inflammation, diabetes, and stress exposure. These factors have been associated with an increased susceptibility to both conditions, indicating a potential correlation between oral health and cognitive well-being [32]. Therefore, another important factor in the pathogenesis of both conditions is age. Elderly patients are more susceptible to developing periodontitis and Alzheimer’s disease than the young [33]. Understanding and addressing these shared risk factors could be crucial in developing preventive strategies targeting both periodontitis and Alzheimer’s disease [30].

The aim of this review is to present the recent aspects of the correlation between periodontitis and AD that have been extensively studied, revealing potential mechanisms linking oral health to neurodegeneration.

## 2. Pathogenesis of Periodontal Diseases

The pathogenesis of periodontal inflammation is not solely related to bacterial–inflammatory interactions but is multi-layered. Significant changes also occur in the transcriptomes (mRNA), proteomes, and metabolomes of gingival fluid and saliva. The gingival fluid itself, as an inflammatory exudate rich in proteins, remains a valuable source of nutrients that promote the development and maturation of the bacterial biofilm. Periodontal pockets constitute a source of transcriptomic, proteomic, and metabolomic alterations in saliva. The culmination of these interactions is overseen by genetic and environmental risk factors, which could significantly alter genetic signaling through epigenetic processes [1,2]. Improper oral hygiene is perceived as the leading cause of bacterial biofilm formation on tooth surfaces. Secondary contributing factors are subgingival calculus, dental cavities, especially those proximal to the gum line, anatomical variations like dental anomalies, incorrect frenula attachment, and failure in both conservative dental treatment and fixed or removable prosthetic restorations, identified as iatrogenic niches [1,2].

Socransky et al. were pioneers in proposing bacterial complexes that are notably more prevalent in periodontal pocket biofilms, based on DNA–DNA hybridization and specific antibody levels against bacteria isolated from DNA [34,35]. The red complex, including *Porphyromonas gingivalis*, *Tannerella forsythia*, and *Treponema denticola*, is associated with a severe status of periodontitis. Recent studies using 16S rRNA gene sequencing in inflamed periodontal tissues exhibited increased levels of *Porphyromonas gingivalis*, *Porphyromonas endodontalis*, *Prevotella intermedia*, *Tannerella forsythia*, *Treponema denticola*, *Treponema socranskii*, *Treponema maltophilum*, *Treponema lecytinolyticum*, *Selenomonas sputigena*, *Parvimonas micra*, *Peptostreptococcus saphenum*, and *Fretibacterium fastidiosum* [36].

The pathogenic actions of various periodontal bacteria such as *Porphyromonas gingivalis*, *Aggregatibacter actinomycetemcomitans*, *Tannerella forsythia*, *Treponema denticola*, and *Campylobacter rectus* play a significant role in dysregulating the immune response and contributing to periodontal disease development [37,38,39].

*Porphyromonas gingivalis*, through the secretion of specific peptidases (gingipains) can dysregulate the immune–inflammatory response by degrading important immunological factors like IgG, IgA, complement components, and defensins. It activates matrix metalloproteinases (MMPs), promotes the release of pro-inflammatory mediators and prostaglandins, inhibits protease inhibitors, reduces receptor expression, suppresses the respiratory burst of phagocytes, and uses membrane proteins like RagA and RagB, which could potentially be associated with antibiotic resistance and are involved in bacterial growth and the activation of proinflammatory mediators. Additionally, it inhibits the biosynthesis of IL-8, which impairs chemotaxis [40,41].

*Aggregatibacter actinomycetemcomitans* produces leukotoxins that induce apoptosis in leukocytes [42,43,44]. It also secretes immunosuppressive factors, inhibits and degrades IgG, and penetrates keratinocytes, impairing their function. Moreover, it employs cytolethal toxins that inhibit cell growth cycles [45].

*Tannerella forsythia* produces karylysin, a metalloproteinase that breaks down TNF and inhibits complement, and a novel bacterial serpin called miropin, which is a serine protease that inhibits neutrophil degranulation [46,47].

*Treponema denticola* presents the ability to inhibit the complement cascade and degrade IgG and IgA, and exhibits resistance to defensins, modulating the host response via Toll-like receptor 2 (TLR2). It also releases an immunosuppressive factor that inhibits macrophage activity [48].

*Campylobacter rectus* utilizes leukotoxins as part of its pathogenic ability [49].

These various bacterial actions contribute to the persistence of periodontitis by evading the host immune response, promoting inflammation, and disrupting normal cellular functions, collectively contributing to the progression of periodontal disease [37,38,39].

## 3. Pathogenesis of Alzheimer’s Disease

The amyloid cascade hypothesis, primarily presented by Hardy et al. in 1992, cannot sufficiently explain the neuronal degeneration in AD for multiple reasons [50]. The buildup and deposition of beta-amyloid has recently been recognized as an antimicrobial response. Beta-amyloid demonstrates antimicrobial efficacy against eight prevalent and clinically significant microorganisms in mice and worm models [51,52]. The potential entry of peripheral amyloid into the brain might take place at circumventricular organs, where endothelial cells lack tight junctions. Alternatively, this transport could occur at the blood–brain barrier, facilitated by the binding of peripheral amyloid to the receptor for advanced glycation end products. An imbalance between its influx and efflux mechanisms could lead to the accumulation of amyloid in the brain [53].

Patients with AD exhibit elevated levels of the receptor for advanced glycation and product microvessels in their brain, while concurrently displaying reduced levels of low-density lipoprotein receptor-related protein 1 and ATP-dependent efflux transporter P-glycoproteins. This perturbation in the equilibrium of these crucial transport mechanisms may contribute to the accumulation of amyloid in the brain, a characteristic feature of the disease [54].

Proteins associated with AD have a capacity to activate the complement system, leading to the recruitment of activated glial cells, including astrocytes and microglia to the amyloid plaque. These glial cells then release proinflammatory cytokines and other toxic mediators, which could contribute to neuronal degeneration in the context of AD [55,56].

In the inflammation hypothesis, a crucial role in the pathogenesis of AD is played by immune–inflammatory processes occurring in the brain [57]. This inflammation is believed to be a key factor in the disruption of synaptic function, the emergence of brain abnormalities, the progression of neurodegenerative changes, and the eventual onset of cognitive impairment. This suggests that brain inflammation is a pivotal element in the cascade of events leading to cognitive decline and neurodegenerative diseases [55,56,57,58].

In research conducted on animal models, the introduction of lipopolysaccharides, a highly potent inflammatory stimulant, through peripheral administration led to several significant outcomes, including the activation of astrocytes and the production of amyloid β42 in both the hippocampus and cerebral cortex. These effects were attributed to an increase in the expression of amyloid precursor protein, along with heightened activities of β- and γ-secretases, as well as tau phosphorylation [59,60].

Furthermore, it was observed that amyloid β could trigger the production of cytokines by glial cells. The administration of anti-inflammatory drugs was found to effectively mitigate these effects, highlighting the interplay between inflammation and the production of Alzheimer’s-associated pathological proteins in the brain. Amyloid β, a hallmark protein in AD, presents the ability to activate glial cells, leading to an increased production of cytokines, which are signaling molecules involved in the immune response. This heightened cytokine production contributes to the inflammatory environment observed in Alzheimer’s-affected brains [55,61,62].

The pathogenesis of AD is often marked by calcium dysregulation. The activity of calcium-related receptors might be affected by amyloid β. Changes in calcium homeostasis are detected in the early stages of AD and are accompanied by changes in calcium-dependent proteases. This might lead to neuron structural damage, cell necrosis and dysfunction, the deposition of amyloid β, abnormal phosphorylation of tau protein, and abnormal synaptic plasticity in the brain. Trypsin-like proteases activate the receptors that facilitate the triggering of adherence molecules linked to tyrosine kinase calcium-dependent receptors as a part of the neurotransmitter pathways supporting brain organization and general health [63].

Clinical studies provide additional proof of the involvement of inflammation in AD [24,55,64,65,66]. Elevated levels of various inflammatory markers in individuals with AD compared to control subjects were observed. These markers include blood interleukin-1beta, interleukin-2, interleukin-6, interleukin-18, α-1 antichymotrypsin, and C-reactive protein. Such findings underscore the association between AD and an inflammatory response, suggesting a potential role for inflammation in the development or progression of the condition [67]. In contrast to the brains of younger individuals, within aged and diseased brains where microglia have undergone prior priming, there is an amplified inflammatory response that hastens the onset of cognitive decline [24,32].

Tau protein, discovered in 1970, is a vital microtubule-interacting protein crucial for stabilizing the neuronal cytoskeleton [68,69]. The tau structure undergoes various posttranslational modifications, including acetylation, glycosylation, glycation, methylation, truncation, nitration, ubiquitination, and phosphorylation [55]. The phosphorylation process takes precedence due to its pivotal role in pathological aggregate formation, associated with a range of neurological diseases, including AD or “tauopathies” [70,71]. In AD brains, around 45 of the 85 phosphorylation sites on tau protein are affected [56]. Early phosphorylation events at specific serine residues, namely Ser199, Ser202/205, and Ser262, disrupt tau’s association with microtubules, leading to altered cellular functions like dysregulated axonal growth and vesicle transport [71,72,73]. Subsequent phosphorylation at other serine residues, such as Ser396, is implicated in AD progression [71].

The process of acetylation prevents the binding of ubiquitin to tau protein. Ubiquitin is a protein involved in the labeling of other proteins for degradation. When acetylation occurs on tau protein, it hinders the ubiquitin-mediated degradation process, potentially leading to increased cytosolic tau levels. Elevated cytosolic tau levels can make the protein more susceptible to aggregation [74]. O-GlcNAcylation is a process in which a sugar molecule (O-linked N-acetylglucosamine) attaches to proteins, including tau protein. Therefore, O-GlcNAcylation seems to present a protective effect against tau-induced pathology. In AD, there is a reduction in the levels of O-GlcNAcylated tau compared to healthy individuals, suggesting a potential link between reduced O-GlcNAcylation and disease progression [75]. Consequently, targeting these diverse posttranslational modifications presents promising ways for preventing tau aggregation and restoring normal protein function.

ApoE serves as a primary cholesterol carrier and is prominently expressed in astrocytes, with lesser expression in microglia. It plays a significant role in mediating the transport and delivery of lipids between different cell types [76]. ApoE plays a crucial role in Aβ aggregation and clearance, influencing senile plaque formation and the development of AD [75,77]. Numerous studies, including clinical, epidemiological, and genetic research, have established an association between ApoE genotypes and AD. The ε4 allele of ApoE, identified through genome-wide association studies, stands out as one of the most potent genetic risk factors for AD. This allele is significantly enriched in AD patients and correlates with an increased Aβ plaque load in the brain, greater brain atrophy, and an earlier onset and accelerated progression of the disease [78,79]. Furthermore, Aβ deposition and plaque formation are notably more prevalent in carriers of the ApoE ε4 allele compared to non-carriers [80,81]. ApoE ε4 carriers also exhibit lower Aβ42 levels in cerebrospinal fluids and higher PiB-positive imaging, indicating a distinct association with altered Aβ metabolism and deposition patterns [82].

## 4. Materials and Methods

A literature search for relevant papers indexed in the literature from 2017 to 2023 was conducted using the PubMed, Scopus, and Google Scholar databases. In our paper, we included clinical trials and research conducted on animal models concerning the correlation between periodontitis and Alzheimer’s disease development. Keywords included: “periodontitis and Alzheimer’s Disease”, “*Porphyromonas gingivalis* and Alzheimer’s disease”, “lipopolysaccharide and Alzheimer’s disease”, and “pathogenesis of Alzheimer’s disease”. We also screened the references of the systematic reviews and meta-analyses to identify additional, original studies that were not found in our prior search.

## 5. Correlation between Alzheimer’s Disease and Periodontitis

Clinical studies comparing the possible associations and occurrence of periodontitis among individuals with and without Alzheimer’s disease have suggested a potential correlation between these conditions.

### 5.1. The Correlation between Periodontal Bacteria and Alzheimer’s Disease

Brains of individuals with AD subjected to autopsy have substantiated the existence of certain microorganisms, including *Actinomycetes* [83,84], *P. gingivalis* [85,86], *Helicobacter pylori*, *Chlamydia pneumoniae* [87], Herpes Simplex type 1 virus (HSV1), specific species of oral and non-oral spirochetes [88], as well as particular fungi [89].

Research conducted by Lee et al. indicated a notably higher occurrence of dementia among individuals with periodontitis, especially those over 80 years old [90]. Similarly, Demmer et al. observed increased risks of dementia or mild cognitive impairment associated with severe periodontal conditions, particularly in younger participants [91]. Choi et al. revealed that periodontitis was linked to a higher risk of dementia and Alzheimer’s disease, even after accounting for lifestyle factors [92].

Research indicates an approximately 1.7-fold higher risk of developing AD with exposure to periodontitis [93,94]. Moreover, individuals with mild to severe periodontitis displayed reduced cognitive function compared to a healthy control group [95]. AD patients exhibited elevated serum TNF-α levels and increased serum antibodies to various periodontal pathogens like *P. gingivalis*, *A. actinomycetemcomitans*, *T. forsythia*, *F. nucleatum*, and *P. intermedia*, suggesting potential links between periodontal disease and cognitive decline [96,97].

Studies conducted by Ilievski et al. have established a correlation between periodontal disease and the accumulation of brain amyloid-beta (Aβ) in humans [98]. For example, in animal models, infection with *P. gingivalis* led to neurodegeneration and increased extracellular Aβ42 production [38]. LPS derived from *A. actinomycetemcomitans* was found to enhance neuroinflammation, leading to the accumulation of Aβ42 [99].

The conducted research emphasizes the presence of *P. gingivalis* and its virulence factors, particularly gingipains, in AD patients’ brains compared to control groups. Mouse studies indicated that *P. gingivalis* migration from the mouth to the brain resulted in increased Aβ42 production and significant neurotoxic effects, which were mitigated by gingipain inhibitors [39,100]. The presence of *P. gingivalis* DNA in the brains of ApoE-null mice suggested a crucial role of the ApoE genotype in neuroinflammation and the potential facilitation of *P. gingivalis* colonization in the brain [101]. Heightened systemic inflammation observed in periodontitis with the absence of a functional ApoE protein may result in blood–brain barrier (BBB) deterioration, allowing periodontal pathogens to enter the systemic circulation, potentially leading to brain invasion [25,102]. Notably, the conducted studies have identified the presence of periodontal pathogens, particularly *P. gingivalis*, in the brain tissue of individuals diagnosed with Alzheimer’s disease [85]. This suggests that *P. gingivalis* may present the capability to breach the BBB; however, the specific mechanisms explaining this phenomenon remain unknown. Additionally, it is crucial to explore the potential contribution of other periodontal pathogens in causing bacteremia and their role in cognitive decline. This association is further underscored by the observed increase in systemic inflammation and heightened activity of microglia, which are specialized immune cells in the brain, along with heightened activation of glial cells. These cellular responses are likely influenced by the presence of periodontal bacteria, particularly the virulence factors known as gingipains produced by *P. gingivalis* [101,103,104,105].

Tang et al. suggested that *T. denticola* induces tau hyperphosphorylation, a characteristic feature of AD, by activating hippocampal neuroinflammation. However, their study did not find evidence for a combined effect of *T. denticola* and *P. gingivalis* on tau phosphorylation [106].

The conducted studies utilizing mice models revealed a potential association between oral pathogens, such as *P. gingivalis* and *T. denticola*, and the onset or progression of Alzheimer’s disease-like symptoms. According to Dominy et al., mice orally infected with *P. gingivalis* displayed colonization of the brain and increased production of Aβ 1–42, a component linked to amyloid plaques in AD. Gingipains, compounds produced by *P. gingivalis*, were proven to be neurotoxic both in vitro and in vivo. The use of small-molecule inhibitors targeting gingipains reduced the bacterial load, blocked Aβ 1–42 production, lessened neuroinflammation, and rescued neurons in the hippocampus. This suggests that gingipain inhibitors might hold promise for treating *P. gingivalis*-related brain infections and neurodegeneration associated with AD [86]. In a study conducted by Wu et al., oral infection of mice with *T. denticola* led to alveolar bone loss and neuronal apoptosis. The authors of that study proposed that this mechanism might be mediated by amyloid-β (Aβ) through the intrinsic mitochondrial pathway [107].

Perivascular spaces constitute a potential pathway for the spread of bacteria in the context of periodontitis [108]. The systemic circulation through these spaces allows bacteria and their products to directly access the brain [109]. This mechanism provides a means for the dissemination of bacteria associated with periodontitis, allowing them to reach the brain and potentially contribute to the development or exacerbation of neurological issues [96]. The circumventricular organs (CVO) serve as entry points for substances that normally do not cross the BBB, enabling them to induce changes in brain function. In contrast to the tight junctions found in the BBB, the capillary endothelial cells in the blood vessels of CVO regions have more permeable junctions, potentially providing a route for the entry of bacteria into the brain [109,110].

According to the olfactory hypothesis, the olfactory tract may potentially serve as a pathway for pathogens to infiltrate the brain, thereby potentially triggering the production of amyloid beta and neurofibrillary tangles. Interestingly, neurofibrillary tangles in AD are caused by hyperphosphorylated tau protein [111], and dysregulated phosphate and hyperphosphatemia are associated with bone mineral disorders in periodontal disease and tumor genesis in cancer. Therefore, phosphate toxicity is suggested as a plausible mediating factor in the correlation of AD with periodontitis and oral cancers [112]. This theory highlights the involvement of both the trigeminal and olfactory nerves as conduits for various periodontal pathogens to reach the central nervous system (CNS), effectively circumventing the BBB [113,114,115]. The identification of oral treponemes in the trigeminal ganglia has prompted researchers to explore this particular route as a potential means for oral microorganisms to access the brain [116].

Given the essential role of gingipains in the survival of *P. gingivalis*, inhibiting them could offer a potential mechanism for treating periodontitis. If *P. gingivalis* indeed serves as a link between periodontitis and AD, inhibiting gingipains should theoretically have an impact on AD pathology as well [40,41,86,117].

Dominy et al. conducted a study where they developed a series of small molecule gingipain inhibitors and assessed their impact on gingipain neurotoxicity in both wild-type mouse brains and an in vitro system utilizing a neuronally-differentiated neuroblastoma cell line. When mice were pre-treated with gingipain inhibitors, their hippocampal neurons were shielded from the neurotoxic effects caused by directly injecting gingipain into the hippocampus [83]. Moreover, the gingipain inhibitors demonstrated protective effects on cultured cells against the toxic impacts of *P. gingivalis*, unlike antibiotics such as moxifloxacin and doxycycline or semagacestat [39,40,86,117,118]. Therefore, when a gingipain inhibitor was orally administered to mice with established brain infection by *P. gingivalis*, it resulted in a reduction in the abundance of *P. gingivalis* DNA in the brain. Additionally, levels of beta-amyloid and the inflammatory mediator TNFα were decreased. Furthermore, the administration of gingipain inhibitors alleviated the neurotoxic effects associated with *P. gingivalis* infection, leading to a significant increase in the number of hippocampal neurons in the brain [86,117,119].

### 5.2. The Impact of Lipopolysaccharides on Alzheimer’s Disease Development

Another significant factor to consider is the impact of lipopolysaccharides (LPS), which are components of bacterial cell membranes, on the brain. LPS molecules can influence the brain’s immune response. Glial cells express pattern recognition receptors (PRRs), such as toll-like receptors 2 and 4 (TLR-2/4), which can detect pathogen-associated molecular patterns (PAMPs) and subsequently trigger antibacterial responses [6,120,121]. *P. gingivalis* produces two heterogeneous forms of lipopolysaccharides: O-LPS and A-LPS [122]. The A-LPS form may be of particular interest due to its potential role in the pathogenesis of Alzheimer’s disease [86,123]. Lipopolysaccharide (LPS), prevalent in the brains of AD patients, was found co-localized with amyloid plaques, suggesting a potential association between LPS and AβPs (Aβ40/42) [124,125]. Injecting LPS in mice induced microglial activation and led to neuroinflammation, potentially exacerbating the expression and processing of amyloid precursor protein (APP) and increasing Aβ40/42 levels in neurons [59,60]. 

It was discovered that prolonged systemic exposure to lipopolysaccharide from *P. gingivalis* leads to AD-like features, such as microglia-driven neuroinflammation, intracellular accumulation of beta-amyloid in neurons, and impaired learning and memory functions in middle-aged mice, dependent on Cathepsin B (CatB). This suggests that targeting CatB may be a potential therapeutic strategy for preventing cognitive decline associated with periodontitis in AD [126]. Lee et al. observed that peripheral administration of lipopolysaccharides (LPS), a potent inflammation-triggering substance, activated astrocytes. This activation led to increased production of amyloid β42 in specific brain regions by upregulating amyloid precursor protein expression, enhancing β- and γ-secretase activities, and promoting tau phosphorylation, which is associated with AD [127]. Additionally, the capacity of amyloid β to trigger glial cells and increase cytokine production was highlighted [60]. Jaeger et al. found that peripheral administration of LPS resulted in elevated amyloid β levels within the brain by altering its transport across the blood–brain barrier. This alteration was characterized by an increased influx and decreased efflux of amyloid β, decreased expression of amyloid β transporter proteins, and heightened levels of certain cytokines in the bloodstream, such as IL-6, IL-8, IL-10, IL-13, and monocyte chemoattractant protein-1 [128]. Leptomeningeal cells, which act as a critical interface between the brain and the immune system, respond to peripheral inflammatory molecules, including lipopolysaccharides. Consequently, a signal from the periphery is relayed to the brain, where it amplifies and contributes to increased brain inflammation [109,122,128,129,130]. Furthermore, lipopolysaccharides were found in close proximity to amyloid β42 within amyloid plaques and in association with amyloid β40 around vessels in the brain tissues of individuals diagnosed with AD [85,86,131].

Periopathogens are both extracellular and intracellular bacteria presenting a capacity to aggregate in both environments. This aggregation is dependent on saliva properties (e.g., agglutinin, proline-rich proteins, and others) and adherent surface molecules such as phosphatidylserine, a variety of glycoprotein-lectin-sialylated molecules, and inherent surface molecules identified by their Gram staining, such as teichoic acid or LPS. Intracellular periopathogens demonstrate a need for carbohydrate O-glycosidic linkages of monosaccharides and disaccharides: glycan–glucan bridges. However, this organization accompanies the enzyme activity of α-D-glucosidase and N-acetyl-β-D glucosaminidase. These enzymes hydrolyze oral epithelial cell oligosaccharides, along with proteoglycans. This degradative function can extend to heparan sulfate and HSPG receptor signaling used by oral viruses such as herpesviruses and SARS-CoV-2. Associated with this action is a triggering of proteases (ADAMs: disintegrins and metallo-proteinases). It should be noted that the zinc-metallo-peptidases (serine endopeptidases), karyilysin, and other metallo-peptidases released by periopathogens function like furin, affecting viral adherence and expanding inflammation from the oral cavity to the brain tissue. Bacterial plasmids have an impact on periopathogen virulence, affecting the range of activity stated. In addition, there are bacterial derived cysteine proteases that also influence the development of brain disorders [132].

### 5.3. The Correlation between Inflammatory Mediators and Alzheimer’s Disease

Inflammation has been observed to affect the integrity of the BBB, leading to increased permeability to cytokines and microbial components like LPS. This heightened permeability may contribute to the entry of inflammatory substances into the brain, potentially influencing neurodegenerative processes [61,129]. The distribution of cytokine transporters within the brain varies and can be influenced by environmental factors, including systemic inflammation and infection. There is also evidence of neuronal pathways that facilitate the passage of molecules originating in the oral cavity [113,114]. Ilievski et al. highlighted that chronic oral application of a periodontal pathogen resulted in brain inflammation, neurodegeneration, and the production of amyloid beta in wild-type mice, indicating a potential connection between oral pathogens and AD-related symptoms [98]. Wang et al. suggested the involvement of interleukin-1β (IL-1β) and tumor necrosis factor-alpha (TNF-α) in modulating the risk of both periodontitis and AD, further indicating the interplay between oral health and neurodegenerative conditions [133]. Studies conducted by Kornman et al. indicated an escalated local immune-inflammatory response in periodontitis, marked by heightened proinflammatory cytokines such as IL-1, IL-6, and TNF-α [134].

Individuals with AD encounter heightened oral health challenges due to progressive cognitive decline, which could impede regular oral hygiene practices. Systemic inflammation is implicated in neurodegeneration by initiating microglial activation and the subsequent release of pro-inflammatory molecules, potentially fueling Alzheimer’s progression [93,135,136].

The conducted studies suggest that maintaining pathogen-free conditions may delay neurodegeneration in murine models experiencing nerve growth factor (NGF) deprivation [90]. The conducted studies also revealed an association between chronic periodontitis and elevated levels of plasma C-reactive protein (CRP) [137,138]. The role of pathogen-free conditions in slowing neurodegeneration in a mouse model, suggesting a potential link between environmental factors and neurological health, was also highlighted [131]. Elevated levels of inflammatory markers like IL-1β, IL-6, CRP, and others in AD were also detected [67,138]. These markers were associated with an increased risk and progression of the disease. Additionally, markers of brain amyloid β burden were found to correlate with proinflammatory molecules, emphasizing the potential role of inflammation in AD pathology [139]. Moreover, research conducted by Warren et al. linked circulating inflammatory markers with changes in brain structure and function in elderly populations, indicating a potential association between systemic inflammation and alterations in brain health over time [64].

Phosphorylated dihydroceramide (PDHC) lipids, which are produced by *P. gingivalis*, include phosphoethanolamine (PE DHC) and phosphoglycerol dihydroceramide (PG DHC) lipids that mediate cellular effects through Toll-like receptor 2 (TLR2). PDHC lipids are present in human tissues, including gingiva, blood, vasculature tissues, and brain. The distribution of TLR2 receptors in human tissues varies with both the tissue site and disease status, which might suggest that PDHC lipids pose an impact on human disease development [140]. Yamada et al. revealed that in mouse model studies, PGDHC lipid, extracted from *P. gingivalis*, enhanced the secretion of soluble amyloid beta peptide and boosted the expression of amyloid precursor protein in CHO-7WD10 cells. Additionally, it prompted increased hyperphosphorylation of tau protein in human neuronal SH-cells upon exposure to PGDHC. Moreover. PGDHC was found to be an agent contributing to the cellular senescence of SH-SY-5Y cells by generating SASP markers such as beta-galactosidase, cathepsin B, inflammatory cytokines TNF-a and IL-6, and reducing the expression of the senescence protection marker sirtuin-1. These findings suggest that PGDHC might represent a new type of virulence factor derived from bacteria associated with AD. This discovery requires further research aimed at understanding the molecular mechanisms linking periodontitis to AD pathology [141]. It has been observed that certain nonsteroidal anti-inflammatory drugs (NSAIDs) possess the capability to counteract this process [142]. A number of epidemiological studies provided evidence that the prolonged use of nonsteroidal anti-inflammatory drugs can play a beneficial role in lowering the risk of AD. NSAIDs inhibit the excessive production of cytokines, potentially mitigating the neuroinflammatory response associated with amyloid β accumulation. This discovery underscores the potential therapeutic value of anti-inflammatory agents in managing the inflammatory component of AD [143,144].

Of additional importance are the surface periopathogen molecules. They affect immunoglobulin-like receptor activity, which contributes to organizing oral mucosal and neural inflammation, particularly through antigen recognition and T cell differentiation. T cell alterations have also been observed in the intracellular signaling pathway in AD patients, such as changes in the calcium response and hyperactivity to amyloid β stimulation [116]. The periopathogens’ metabolism is also associated with the host mucosa and brain tissues, as products derived from their metabolites and enzymes influence the host’s metabolism and physiology. Significantly lower metabolite concentrations of tryptophan pathway metabolites, including serotonin, 5-hydroxyindoleacetic acid, kynurenine, kynurenic acid, tryptophan, xanthurenic acid, and the kynurenine/tryptophan ratio was observed among AD patients [145]. Neuropilin-1 (NRP1), a transmembrane protein with roles in neuronal development, axonal outgrowth, and angiogenesis is strongly expressed in AD patients. Combined effects on metabolism and physiology also result from the presence of periopathogens [146].

The main results of the novel conducted research are presented in Table 1.

## 6. Study Limitations

The correlation between periodontitis and Alzheimer’s disease development still remains a new phenomenon that is not fully explained. In the conducted research, several different mechanisms were observed and cannot be combined into one universal pathomechanism of the impact of periodontitis on Alzheimer’s disease. The different studies are also characterized by different methodologies, study populations, and examined parameters.

## 7. Conclusions

The presented scientific discoveries collectively highlight the potential association between periodontitis and Alzheimer’s disease, suggesting shared pathological pathways, including systemic inflammation, pathogen presence, and compromised BBB integrity. Further research could unveil therapeutic approaches targeting periodontal pathogens to potentially alleviate AD progression.

## Figures and Tables

**Table 1:** Novel results of research on the correlation between periodontitis and Alzheimer’s disease.

Author	Main Results	Type of Study
Wang et al. (2023) [133]	The involvement of IL-1β and TNF-α is modulating the risk of both periodontitis and AD	Mouse model—periodontitis was induced in mice, IL-1β and TNF-α were also injected into the buccal mandibular vestibule; the alveolar bone loss and brain left hemispheres were examined
Tang et al. (2022) [106]	*T. denticola* induces tau hyperphosphorylation, a characteristic feature of AD, by activating hippocampal neuroinflammation.	Mouse model—mice underwent oral infection with *T. denticola* and tau hyperphosphorylation in the hippocampi was examined
Wu et al. (2022) [107]	Oral infection of mice with *T. denticola* led to alveolar bone loss and neuronal apoptosis	Mouse model—mice underwent infection with *T. denticola* or *P. gingivalis*, then alveolar bone loss and the hippocampus were examined (15 mice)
Lee et al. (2020) [90]	A higher occurrence of dementia was observed among individuals with periodontitis, especially those over 80 years old.	Cohort study—56,018 patients aged ≥50 years with newly diagnosed periodontitis
Demmer et al. (2020) [91]	An increased risk of dementia or mild cognitive impairment associated with severe periodontal conditions, particularly in younger participants, was observed	Cohort study—8275 patients underwent a clinical periodontal examination and neurocognitive testing
Yamada et al. (2020) [141]	PGDHC sphingolipid, extracted from *P. gingivalis*, enhanced the secretion of soluble amyloid beta peptide and boosted the expression of amyloid precursor protein	Culture model—Chinese hamster CHO-7WD10 and SH-SY5Y human neuroblastoma cells were exposed to PGDHC and LPS isolated from *P. gingivalis*. APP, phosphorylated tau, and SASP factors were quantified
Choi et al. (2019) [92]	Periodontitis was linked to higher risks of dementia and Alzheimer’s disease.	Cohort study—the study population consisted of 262,349 participants (periodontal procedures and dementia-related drugs were analyzed)
Dominy et al. (2019) [86]	When mice were pre-treated with gingipain inhibitors, their hippocampal neurons were shielded from the neurotoxic effects caused by directly injecting gingipain into the hippocampus	Mouse model—injection of gingipains into the hippocampus (15 mice)
Ilievski et al. (2018) [98]	A correlation between periodontal disease and the accumulation of brain amyloid-beta (Aβ) was observed.	Mouse model—periodontitis was induced in 10 mice; brain tissues were collected and examined for signs of neuropathology
Wu et al. (2017) [126]	Prolonged systemic exposure to lipopolysaccharide from *P. gingivalis* leads to AD-like features	Mouse model—mice were subjected to systemic exposure to PgLPS; their learning and memory function were assessed; the expression of APP, CatB, TLR2, and IL-1β was analyzed in brain tissues (12 mice)

## Data Availability

Not applicable.
